# Diversity and distribution of vascular plants within the treeline ecotone in Mount Iremel (Southern Urals, Russia)

**DOI:** 10.3897/BDJ.9.e69446

**Published:** 2021-07-13

**Authors:** Marina Trubina, Alexey Nesterkov

**Affiliations:** 1 Institute of plant and animal ecology, UB RAS, Ekaterinburg, Russia Institute of plant and animal ecology, UB RAS Ekaterinburg Russia

**Keywords:** species richness, occurrence, frequency, tracheophytes, mountain ecosystems, elevational gradient, transitional plant communities, understorey vegetation

## Abstract

**Background:**

During the last 100 years, rapid advances of trees towards higher elevations and latitudes have been recorded for various regions worldwide, including the Ural Mountains. Climate warming and tree cover increases can lead to significant changes in the high-mountain vegetation. Direct observations on the vegetation of high-mountain regions provide evidence for an increase in the species diversity of plants at high elevations and changes in the composition of the alpine communities. This study investigated the diversity and distribution of vascular plants within the present-day treeline ecotone in Mount Iremel, the Southern Urals.

**New information:**

The dataset (Trubina and Nesterkov 2021, available from the GBIF network at https://www.gbif.org/dataset/284f1484-10b7-4ef5-87b7-9de1159e6b42) presents the results of an assessment of species richness and frequency of vascular plants at the different elevation levels (from 1203 to 1348 m a.s.l.) and different biotopes (birch-spruce shrub forest, birch-spruce sparse forest and spruce forest with fragments of meadow plant communities) within the treeline ecotone in Mount Iremel, Southern Urals. Observations were carried out at 700 sampling plots with two estimation methods: small-size plot (0.5 × 0.5 m) sampling (672 plots in total) and large-size plot (10 × 10 m) sampling (28 plots). The dataset includes 700 sampling events (= sampling plots), corresponding to 5585 occurrences (vascular plants, mainly identified to species) observed during July 2003. Only occurrences containing plant taxa (occurrenceStatus = present) have been provided. The dataset includes information about distribution and frequency of the Ural endemic species (*Anemone narcissiflora subsp. biarmiensis* (Juz.) Jalas, *Calamagrostis uralensis* Litv., *Cerastium krylovii* Schischk. & Gorczak., *Festuca igoschiniae* Tzvel., *Hieracium iremelense* (Elfstr.) Üksip, *Lagotis uralensis* Schischk, *Pleurospermum uralense* Hoffm.) and the Pleistocene relict species (*Alopecurus magellanicus* Lam., *Bistorta vivipara* (L.) Delarbre, *Cerastium pauciflorum* Stev. ex. Ser., *Pedicularis oederi* Vahl, *Saussurea controversa* DC., *Swertia perennis* L.). The dataset also provides information that can be useful for estimating biodiversity and plant communities composition within the treeline ecotone at a specified time period and contributes to the study of biodiversity conservation in the Ural Region.

## Introduction

High mountain ecosystems and their biota are driven by low-temperature conditions and can be used as indicators for climate warming impacts on natural ecosystems. In the 20th century, high-mountain forests increased in range and their boundaries changed in different regions of the world (Jakubos and Romme 1993, Shiyatov 1993, Woodward et al. 1995, Holtmeier 2009, Peñuelas and Boada 2003), including the Southern Urals (Shiyatov 1983, Moiseev et al. 2004, Hagedorn et al. 2014). This phenomenon is usually attributed to global warming. Direct observations on the vegetation of high-mountain regions also provide evidence for an increase in the species diversity of plants at high elevations and changes in the composition of alpine communities (Grabherr et al. 1994, Keller et al. 2000, Lamprecht et al. 2018, Løkken et al. 2020, *Savage and Vellend 2014*). However, the magnitude and direction of upward-elevational shifts of species distribution vary tremendously amongst species and regions, the geographic range of existing studies is relatively sparse and the opportunities for assessing long-term community change provided by historical biodiversity surveys are relatively rare (Savage and Vellend 2014).

The Ural Mountains spread meridionally for more than two thousand kilometres, from the 50^th^ to the 70^th^ parallel. Thus, they are located within several vegetation zones (tundra, forest-tundra, taiga, broad-leaved forests, forest-steppe and steppe). The high-mountain vegetation of these zones is quite specific (Igoshina 1964, Gorchakovskii 1975). Flora and vegetation of the Southern Ural Mountains (including the Iremel) has been actively studied during the last 100 years (Igoshina 1964, Gorchakovskii 1966, Gorchakovskii 1969, Gorchakovskii 1975, Nikonova et al. 1992, Shurova 1982, Shurova 1983, Sharafutdinov 1983, Shiyatov 1983, Ishbirdin et al. 1996). The present-day flora of Mount Iremel includes 322 species of vascular plants (Shurova 1982), 54 species of liverworts and 152 mosses (Baisheva et al. 2015). Amongst the vascular plants, 15 Ural endemics and ten relict species are presented and 46 species are included in the Red Book of Bashkortostan (Ishbirdin et al. 1996).

Mount Iremel is one of the highest mountains in the Southern Urals. Rapid advances of trees towards higher elevations are well documented (Shiyatov 1983, Moiseev et al. 2004, Hagedorn et al. 2014). Despite the long history of the vegetation studies in this region, assessments of the species richness for the vascular plants at different elevational levels are sporadic and relate either to changes in the species richness for plants with different thermal preferences within a present-day treeline ecotone (Trubina 2006) or to changes in the functional and taxonomic diversity within the treeline ecotone at the single slope (Gazol et al. 2017). Quantitative assessments of the species distribution at the different slopes of this mountain massif have never been conducted. However, due to their geographical location, the mountain tundra and the sub-alpine belt communities of the Southern Urals may be particularly vulnerable to climate warming and the rapid upward movement of trees. Moreover, most of the endemic and relict species of the Urals are extremely vulnerable to environmental changes, as they are associated with high mountain communities and have low population size (Gorchakovskii and Shurova 1982). The dataset includes information about distribution and frequency of the Ural endemic species (*Anemone narcissiflora subsp. biarmiensis* (Juz.) Jalas, *Calamagrostis uralensis* Litv., *Cerastium krylovii* Schischk. & Gorczak., *Festuca igoschiniae* Tzvel., *Hieracium iremelense* (Elfstr.) Üksip, *Lagotis uralensis* Schischk, *Pleurospermum uralense* Hoffm.) and the Pleistocene relict species (*Alopecurus magellanicus* Lam., *Bistorta vivipara* (L.) Delarbre, *Cerastium pauciflorum* Stev. ex. Ser., *Pedicularis oederi* Vahl, *Saussurea controversa* DC., *Swertia perennis* L.). The dataset also provides helpful information for estimating the biodiversity and composition of plant communities within the treeline ecotone at the different slopes and at a specified time period and contributes to the study of biodiversity conservation in the Ural Region.

## Project description

### Title

Diversity and distribution of vascular plants within the treeline ecotone in Mount Iremel (Southern Urals, Russia)

### Study area description

**Figure 1.**
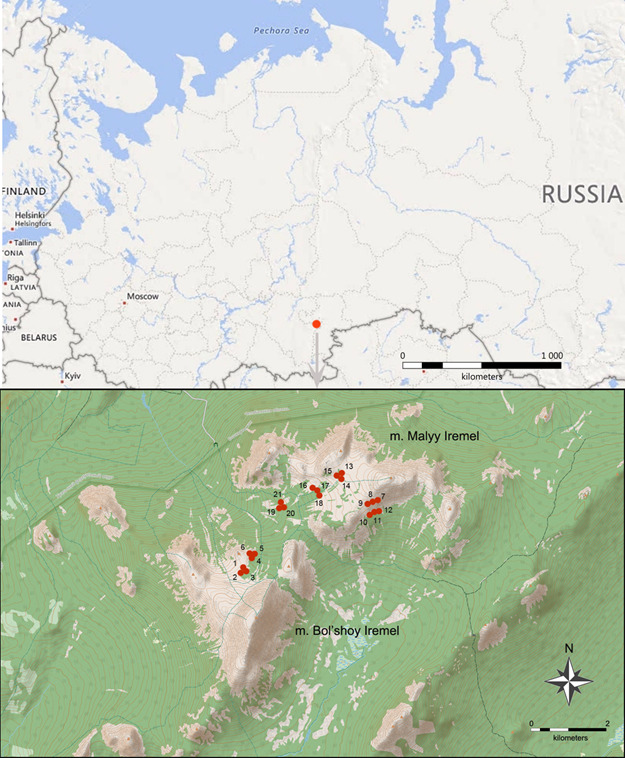
Location of the macro-plots at the slopes of the mountains Bol’shoy and Malyy Iremel, Southern Urals (data from Open Street Map; numbers of the macro-plots correspond to those in the dataset).

**Figure 2.**
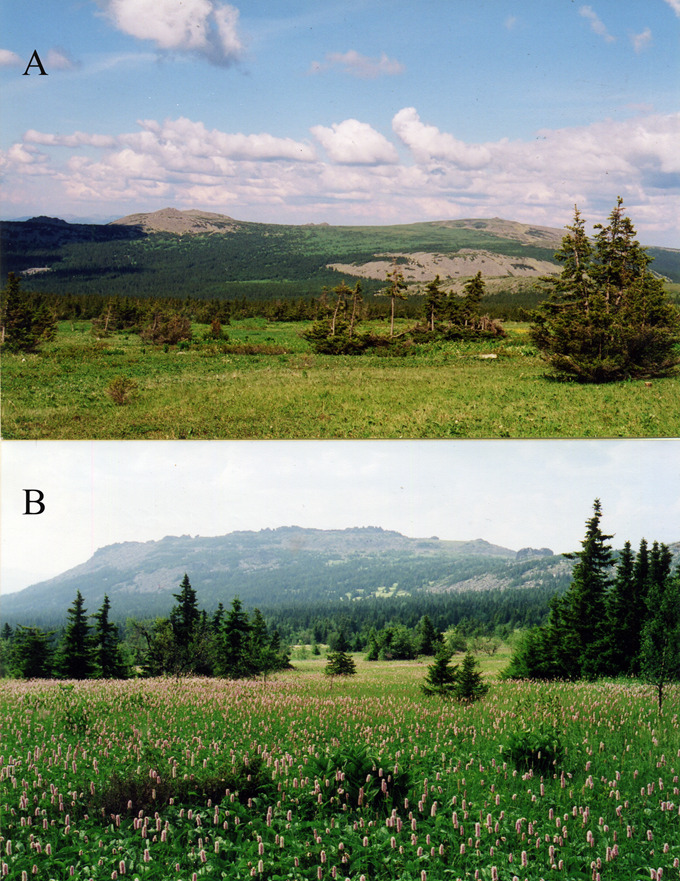
Birch-spruce shrub forest (**A**) and birch-spruce sparse forest; (**B**) at the slopes of mount Malyy Iremel (photos made in 2003).

Mount Iremel is located in the region of the highest elevation of the Southern Urals within the Iremel-Avalyak mountain region (Fig. 1). On the joint base of Mount Iremel, two peaks rise – the second largest one of the Southern Urals, Bol’shoy Iremel (1582 m a.s.l.) and Malyy Iremel (1450 m a.s.l.). The average annual temperature is 0.3°C and the precipitation average is 600–700 mm, which is typical for the northeast of the mountainous part of the Bashkortostan. The mountain forest, subgoltzy (subalpine) and mountain tundra belts are distinguished in the vegetation of Mount Iremel (Gorchakovskii 1975). The subalpine zone extends from 1100–1150 m to 1350–1400 m a.s.l.. Park-type open spruce and spruce–birch forests and tall-herb meadows, dominated by Alpine knotweed (*Persicaria alpina*) and snakeweed (*Persicaria bistorta*), are characteristic for its lower part. In contrast, spruce and birch shrub forests with small-herb meadows prevail in their upper part (Fig. 2) (Nikonova et al. 1992). The range of elevations occupied by the mountain tundra belt is about 200 m. The herb–moss tundra type prevails, but stony, lichen, dwarf shrub–moss and complex tundra types are also presented (Sharafutdinov 1983). Siberian spruce *Picea obovata* and birch *Betula tortuosa* are the main tree species forming the treeline. Currently, Mount Iremel has been assigned the status of a nature park.

## Sampling methods

### Study extent

The study was carried out in the Southern Urals, Russia, within the Iremel mountain group, at the slopes of the mountains Bol’shoy (54.52000°N, 58.84167°E) and Malyy (54.55167°N, 58.89167°E) Iremel. A total of 700 sampling plots (672 plots of 0.5 × 0.5 m and 28 plots of 10 × 10 m) were established in three types of biotopes: 300 plots in a birch-spruce sparse forest, 300 plots in a spruce forest with fragments of meadow plant communities and 100 plots in a birch-spruce shrub forest. The study was completed in July 2003.

### Sampling description

**Figure 3.**
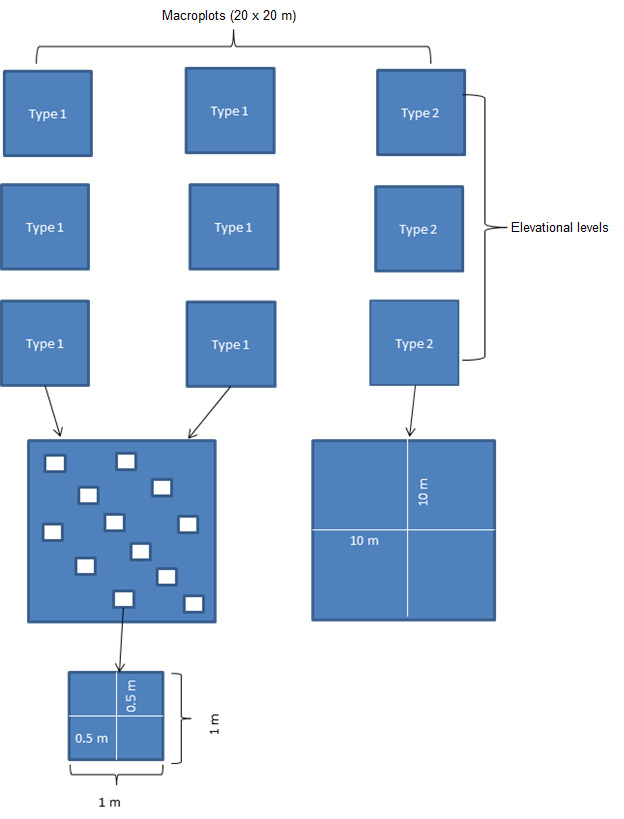
General scheme of an experiment design (Type 1 and Type 2 are the methods for description of the plant species composition).

Vertical transects (Fig. 3) were established within the present-day treeline ecotone at the south-western and southern slopes of mount Malyy Iremel and the north-eastern slope of mount Bol’shoy Iremel (three transects on each slope, nine transects in total). At the southern slope of Malyy Iremel and the north-eastern slope of Bol’shoy Iremel, within each transect, two elevation levels were identified corresponding to biotope types (birch-spruce sparse forest and spruce forest with fragments of meadow plant communities). At the south-western slope of Malyy Iremel, three elevation levels were identified (the corresponding biotopes are birch-spruce sparse forest, birch-spruce shrub forest and spruce forest with fragments of meadow plant communities). Detailed characteristics of the biotopes were published earlier (Trubina 2006).

Three sampling sites, 20 × 20 m (also referred to as macro-plots (= locationID in the dataset, 21 in total), were established within each elevation level. The central points of the sampling sites were positioned at a distance of 50–70 m from each other. Each macroplot was divided into four subplots of 10 × 10 m. At each elevation level, within two macroplots, 12 plots with a size of 1 × 1 m were located (total number 168), within which, in turn, four sampling plots with a size of 0.5 × 0.5 m were placed (total number 672). The small plots with a size of 0.5 × 0.5 m were established for assessment of plant species frequency. Within the third macroplot at each elevation level, the sampling was carried out only in subplots with a size of 10 × 10 m (total number 28).

The sampling process included direct observations and active search to find all vascular plant species in understorey vegetation in sampling plots of two sizes (0.5 × 0.5 m and 10 × 10 m). Sampling effort (time interval for describing all plants in one sampling plot) was approximately 10 minutes for small-sized plots and 50 minutes for large-sized plots. A large number of plots (700 in total) made it possible to take into account the irregularity in distribution of plant species within the study sites. The use of small-sized sampling plots allows obtaining quantitative data (frequency of species) to estimate the species abundance.

### Quality control

Identification of plant species was carried out mainly in the field; specimens with controversial species affiliation were herborised and identified later in a laboratory by specialists from the Institute of Plant and Animal Ecology of the Ural Branch of the Russian Academy of Sciences (IPAE UB RAS). Identification was cross-checked by specialists from the Institute Botanical Garden of the Ural Branch of the Russian Academy of Sciences (IBG UB RAS).

## Geographic coverage

### Description

Sampling plots were founded at an altitude of 1200–1350 m a.s.l. within the present-day ecotone of the upper forest line. At the southern slope of Malyy Iremel and the north-eastern slope of Bol’shoy Iremel, plots were located in two types of biotopes (birch-spruce sparse forest and spruce forest with fragments of meadow plant communities); at the south-western slope of Malyy Iremel in three types of biotopes (birch-spruce shrub forest, birch-spruce sparse forest and spruce forest with fragments of meadow plant communities).

### Coordinates

54.471 and 54.591 Latitude; 58.733 and 59.017 Longitude.

## Taxonomic coverage

### Description

General taxonomic coverage is 1 phylum, 4 classes, 22 orders, 30 families, 59 genera, 70 species of vascular plants.

### Taxa included

**Table:** 

Rank	Scientific Name	
phylum	Tracheophyta	
class	Liliopsida	
class	Magnoliopsida	
class	Pinopsida	
class	Polypodiopsida	
order	Apiales	
order	Araliales	
order	Asparagales	
order	Asterales	
order	Brassicales	
order	Caryophyllales	
order	Dipsacales	
order	Ericales	
order	Fabales	
order	Gentianales	
order	Geraniales	
order	Lamiales	
order	Liliales	
order	Malpighiales	
order	Myrtales	
order	Oxalidales	
order	Pinales	
order	Poales	
order	Polypodiales	
order	Ranunculales	
order	Rosales	
order	Saxifragales	
family	Apiaceae	
family	Asparagaceae	
family	Asteraceae	
family	Brassicaceae	
family	Campanulaceae	
family	Caprifoliaceae	
family	Caryophyllaceae	
family	Crassulaceae	
family	Cupressaceae	
family	Cyperaceae	
family	Dryopteridaceae	
family	Ericaceae	
family	Fabaceae	
family	Gentianaceae	
family	Geraniaceae	
family	Hypericaceae	
family	Juncaceae	
family	Liliaceae	
family	Onagraceae	
family	Orobanchaceae	
family	Oxalidaceae	
family	Plantaginaceae	
family	Poaceae	
family	Polygonaceae	
family	Primulaceae	
family	Ranunuculaceae	
family	Rosaceae	
family	Rubiaceae	
family	Salicaceae	
family	Valerianaceae	

## Traits coverage

### Data coverage of traits

PLEASE FILL IN TRAIT INFORMATION HERE

## Temporal coverage

**Data range:** 2003-7-04 – 2003-7-14.

## Collection data

### Collection name

geobot_Iremel_2003

## Usage licence

### Usage licence

Creative Commons Public Domain Waiver (CC-Zero)

### IP rights notes

This work is licensed under a Creative Commons Attribution (CC-BY) 4.0 Licence.

## Data resources

### Data package title

Diversity and distribution of vascular plants within the treeline ecotone in Mount Iremel (Southern Urals, Russia)

### Resource link

https://doi.org/10.15468/6hsht5

### Alternative identifiers

284f1484-10b7-4ef5-87b7-9de1159e6b42, http://gbif.ru:8080/ipt/resource?r=vascular_plants_iremel_2003

### Number of data sets

1

### Data set 1.

#### Data set name

Diversity and distribution of vascular plants within the treeline ecotone in Mount Iremel (Southern Urals, Russia)

#### Data format

Darwin Core

#### Number of columns

39

#### Description

The dataset presents the results of an assessment of the species richness and frequency of vascular plants at the different elevation levels (from 1203 to 1348 m a.s.l.) and in the different biotopes (birch-spruce sparse forest, birch-spruce shrub forest and spruce forest with fragments of meadow plant communities) in the Iremel mountain group (Southern Urals). Observations were carried out at 700 sampling plots using two estimation methods: small-size plot (0.5 × 0.5 m) sampling (672 plots in total) and large-size plot (10 × 10 m) sampling (28 plots). The dataset includes 700 sampling events (= sampling plots) corresponding to 5585 occurrences (vascular plants, identified mostly to species) and observed during July 2003. Only occurrences containing plant taxa (occurrenceStatus = present) have been provided. The dataset provides valuable information for estimating the biodiversity and composition of plant communities within the treeline ecotone, including the information about distribution and frequency of the Ural endemic species (*Anemone narcissiflora subsp. biarmiensis* (Juz.) Jalas, *Calamagrostis uralensis* Litv., *Cerastium krylovii* Schischk. & Gorczak., *Festuca igoschiniae* Tzvel., *Hieracium iremelense* (Elfstr.) Üksip, *Lagotis uralensis* Schischk, *Pleurospermum uralense* Hoffm.) and the Pleistocene relict species (*Alopecurus magellanicus* Lam., *Bistorta vivipara* (L.) Delarbre, *Cerastium pauciflorum* Stev. ex. Ser., *Pedicularis oederi* Vahl, *Saussurea controversa* DC., *Swertia perennis* L.).

**Data set 1.** 

Column label	Column description
eventID	An identifier for the set of information associated with an Event (something that occurs at a place and time). May be a global unique identifier or an identifier specific to the dataset.
occurrenceID	An identifier for the Occurrence (as opposed to a particular digital record of the occurrence).
occurrenceStatus	A statement about the presence or absence of a Taxon at a Location.
basisOfRecord	The specific nature of the data record.
samplingProtocol	The name of, reference to, or description of the method or protocol used during an Event.
samplingEffort	The amount of effort expended during an Event.
sampleSizeValue	A numeric value for a measurement of the size (time duration, length, area or volume) of a sample in a sampling event.
sampleSizeUnit	The unit of measurement of the size (time duration, length, area or volume) of a sample in a sampling event.
eventDate	The date-time or interval during which an Event occurred.
habitat	A category or description of the habitat in which the Event occurred.
locationRemarks	Comments or notes about the Location (name of the mountain and the slope where sampling event took place).
year	The four-digit year in which the Event occurred, according to the Common Era Calendar.
month	The ordinal month in which the Event occurred.
country	The name of the country or major administrative unit in which the Location occurs.
countryCode	The standard code for the country in which the Location occurs.
stateProvince	The specific description of the place.
county	The full, unabbreviated name of the next smaller administrative region than stateProvince (county, shire, department etc.) in which the Location occurs.
infraspecificEpithet	The name of the lowest or terminal infraspecific epithet of the scientificName, excluding any rank designation.
locationID	An identifier for the set of location information (data associated with dcterms:Location).
maximumElevationInMetres	The upper limit of the range of elevation (altitude, above sea level), in m a.s.l
verbatimElevation	The original description of the elevation (altitude, above sea level) of the Location (number of the elevational level).
decimalLatitude	The geographic latitude (in decimal degrees, using the spatial reference system given in geodeticDatum) of the geographic centre of a Location.
decimalLongitude	The geographic longitude (in decimal degrees, using the spatial reference system given in geodeticDatum) of the geographic centre of a Location.
geodeticDatum	The ellipsoid, geodetic datum or spatial reference system (SRS) upon which the geographic coordinates given in decimalLatitude and decimalLongitude are based.
coordinateUncertaintyInMetres	The horizontal distance (in metres) from the given decimalLatitude and decimalLongitude describing the smallest circle containing the whole of the Location. Leave the value empty if the uncertainty is unknown, cannot be estimated or is not applicable (because there are no coordinates). Zero is not a valid value for this term.
ownerInstitutionCode	The name (or acronym) in use by the institution having ownership of the object(s) or information referred to in the record.
scientificName	The full scientific name, with authorship and date information, if known.
scientificNameAuthorship	The authorship information for the scientificName formatted according to the conventions of the applicable nomenclaturalCode.
kingdom	The full scientific name of the kingdom in which the taxon is classified.
phylum	The full scientific name of the phylum or division in which the taxon is classified.
class	The full scientific name of the class in which the taxon is classified.
order	The full scientific name of the order in which the taxon is classified.
family	The full scientific name of the family in which the taxon is classified.
genus	The full scientific name of the genus in which the taxon is classified.
specificEpithet	The name of the first or species epithet of the scientificName.
identificationRemarks	Comments or notes about the Identification.
taxonRank	The taxonomic rank of the most specific name in the scientificName.
recordedBy	A list (concatenated and separated) of names of people, groups or organisations responsible for recording the original Occurrence.
identifiedBy	A list (concatenated and separated) of names of people, groups or organisations who assigned the Taxon to the subject.

## Additional information

See Trubina and Nesterkov (2021).
